# Target-aware transformer tracking with hard occlusion instance generation

**DOI:** 10.3389/fnbot.2023.1323188

**Published:** 2024-01-10

**Authors:** Dingkun Xiao, Zhenzhong Wei, Guangjun Zhang

**Affiliations:** Key Laboratory of Precision Opto-Mechatronics Technology, Ministry of Education, School of Instrumentation and Opto-Electronics Engineering, Beihang University, Beijing, China

**Keywords:** visual tracking, transformer, occlusion, instance generation, target-aware, deep learning

## Abstract

Visual tracking is a crucial task in computer vision that has been applied in diverse fields. Recently, transformer architecture has been widely applied in visual tracking and has become a mainstream framework instead of the Siamese structure. Although transformer-based trackers have demonstrated remarkable accuracy in general circumstances, their performance in occluded scenes remains unsatisfactory. This is primarily due to their inability to recognize incomplete target appearance information when the target is occluded. To address this issue, we propose a novel transformer tracking approach referred to as TATT, which integrates a target-aware transformer network and a hard occlusion instance generation module. The target-aware transformer network utilizes an encoder-decoder structure to facilitate interaction between template and search features, extracting target information in the template feature to enhance the unoccluded parts of the target in the search features. It can directly predict the boundary between the target region and the background to generate tracking results. The hard occlusion instance generation module employs multiple image similarity calculation methods to select an image pitch in video sequences that is most similar to the target and generate an occlusion instance mimicking real scenes without adding an extra network. Experiments on five benchmarks, including LaSOT, TrackingNet, Got10k, OTB100, and UAV123, demonstrate that our tracker achieves promising performance while running at approximately 41 fps on GPU. Specifically, our tracker achieves the highest AUC scores of 65.5 and 61.2% in partial and full occlusion evaluations on LaSOT, respectively.

## 1 Introduction

Visual tracking is a fundamental task in computer vision and is applied in many fields such as virtual reality, intelligent transportation systems, and unmanned aerial vehicles (Abbass et al., 2021; Marvasti-Zadeh et al., 2021). Given the tracking target in the first frame, the tracking task aims to estimate the bounding box of the target in the rest of the video sequence (Li P. et al., 2018). The difficulty of tracking tasks comes from the rapid appearance change of the target and the complexity of the tracking scene, such as occlusion, illumination angle, and background distraction (Jiao et al., 2021).

In the past few years, most advanced trackers have followed the structure of the Siamese network, which extracts template and search features through parallel backbones and learns the relation between them to get the final tracking results (Javed et al., 2022). Recently, transformers (Vaswani, 2017) have drawn a lot of attention in computer vision (Han et al., 2022; Khan et al., 2022). Some trackers apply transformers in their architecture, which has greatly improved the feature utilization and learning ability of the network. These algorithms have demonstrated top-of-the-line performance on most datasets.

However, their performance is still unsatisfactory in occlusion scenes. Especially when the occlusion object is similar to the target, the tracker is easily influenced and may cause the bounding box to shift toward the occlusion object, ultimately leading to tracking failure. This kind of circumstance is referred to as a hard occlusion. Most trackers are designed for general circumstances. They assume that the search images contain sufficient appearance information about the target and directly predict the center position and scale of the target. The lack of occlusion samples during the training process makes it difficult for the tracker to learn how to handle occluded targets and recognize the incomplete target appearance feature. Therefore, when the target is occluded, the tracking accuracy of the tracker will significantly decrease, leading to bounding box drift or even tracking failure.

To solve the mentioned problem, we propose target-aware transformer tracking with hard occlusion instance generation (TATT), which comprises a target-aware transformer network and a hard occlusion instance generation module. The proposed target-aware transformer network employs an encoder-decoder structure. The encoder makes global interaction between the template feature and search feature, while the decoder exploits reliable target information in the template and generates multiple spatial attention maps to enhance the unoccluded parts of the target in the search features. It directly predicts the boundary between the target region and the background to produce a bounding box, minimizing the impact of occlusion. The hard occlusion instance generation module selects an image patch similar to the target from several adjacent frames and randomly masks the target area in the search image to generate occlusion training samples. It has a fast computation speed without increasing offline training complexity and can offer hard occlusion samples that are close to natural scenes. This method helps the network learn to recognize incomplete target appearance information and improve its ability to distinguish the target.

**Figure 1:**
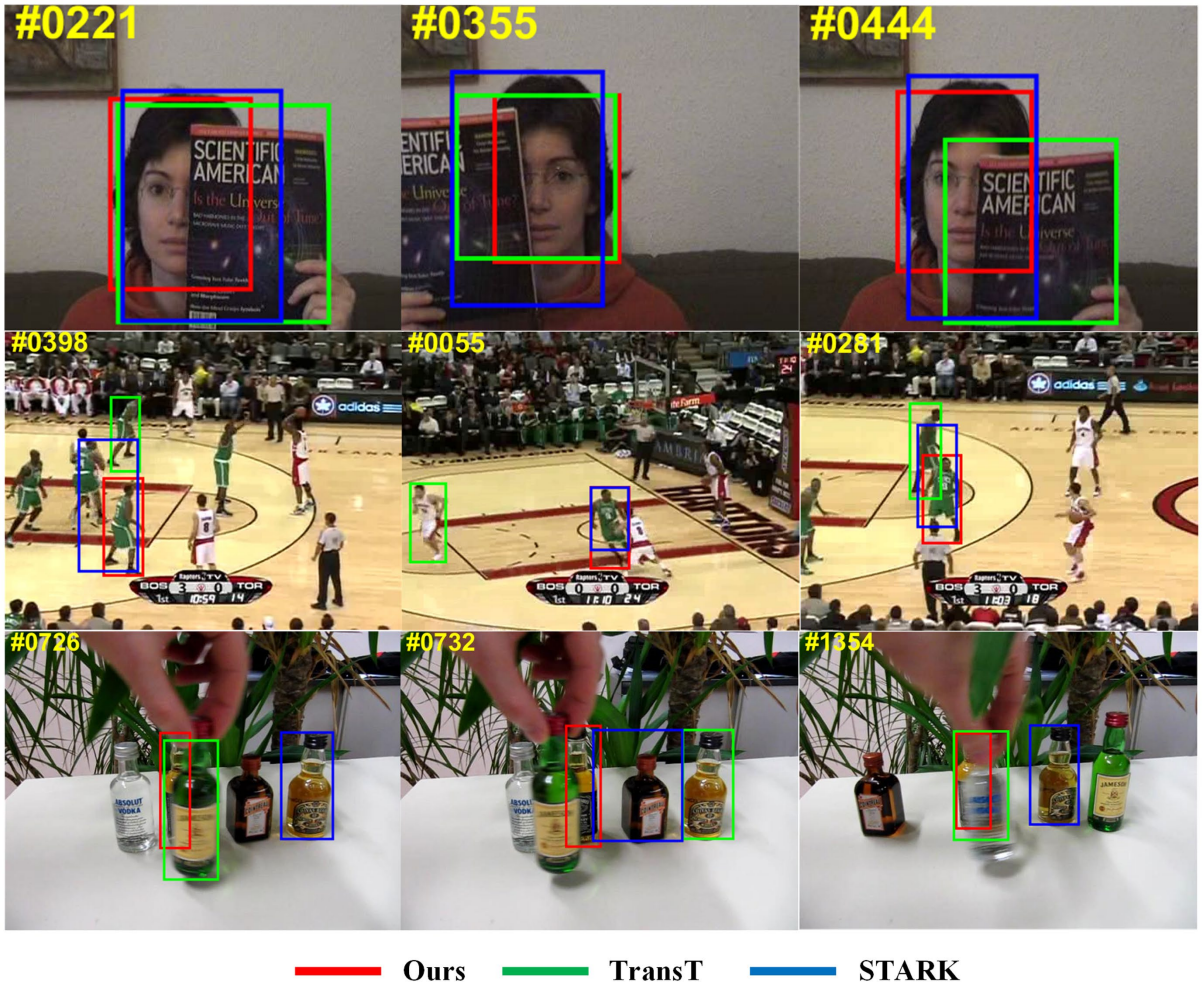
Comparison of the tracking results among ours and TransT, STARK. Our results show better performance in occlusion.

Figure 1 illustrates that our tracker performs better in occlusion scenes compared with TransT and STARK. When an occluding object covers part of the tracked target in the first row of images, our tracker is capable of recognizing the unoccluded portion of the target and predicting its position and scale. In particular, in the scenario of a similar occluding object, as shown in the second and third rows of the results, TATT demonstrates a strong recognition ability toward the target, still being able to lock onto it. On the other hand, the other two algorithms fail to fully cope with occlusion scenarios, resulting in the tracking box shifting toward similar interfering objects in the background, ultimately leading to tracking failure. Our tracker achieves outstanding evaluation results on five benchmarks, including LaSOT (Fan et al., 2019), TrackingNet (Müller et al., 2018), Got10k (Huang et al., 2019), OTB100 (Wu et al., 2013), and UAV123 (Mueller et al., 2016).

## 2 Related work

In recent years, Siamese-based trackers have become the mainstream algorithm in visual tracking (Li P. et al., 2018). These trackers adopt a parallel network structure to simultaneously process template images and search images and predict the bounding box of the target by computing the correlation between the two features. SiamFC (Bertinetto et al., 2016) is the pioneer of this series of works. It extracts template and search features through two streams of backbone sharing the same parameters. Then, it computes cross-correlation between the template and search feature at multiple scales and directly predicts the target position through the highest correlation score. SiamRPN (Li B. et al., 2018) applies the RPN from detection to tracking task, which enables it to perform accurate bounding box regression. SiamRPN++ (Li et al., 2019) proposes a data augment method to address the center bias problem during the training process so that it can employ a deeper backbone such as ResNet50. It improves the performance of Siamese-based trackers to a new level. SiamBan (Chen et al., 2022), SiamCar (Guo et al., 2020), SiamFC++ (Xu et al., 2020), and Ocean apply the anchor-free bounding box regression method instead of RPN to obtain more accurate tracking results. SiamMask (Hu et al., 2023) and D3S (Lukezic et al., 2020) integrate object detection and segmentation with Siamese-based architecture, which achieves high accuracy and robustness. SiamAttn (Yu et al., 2020) incorporates self-attention and cross-attention mechanisms, which leads to better object recognition.

Transformer is a self-attention-based network structure that was initially proposed in natural language and widely used in computer vision (Ye et al., 2019; Carion et al., 2020; Touvron et al., 2021). Recently, several trackers that interact with the transformer architecture have shown exceptional accuracy and robustness in most common datasets. TransT (Chen et al., 2021), TrTr (Zhao et al., 2021), and CSWinTT (Song et al., 2022) adopt a transformer cross-attention module to replace the traditional cross-correlation method. This approach can effectively enhance the interaction between template and search features. ToMP (Mayer et al., 2022) constructs a target model with a transformer network and applies it in a correlation filter-based tracker. MixFormer (Cui et al., 2022) and SwinTrack (Lin et al., 2022) propose a backbone network based on transformer, which interacts between the template feature and search feature in the process of feature extraction. CMAT (Wang et al., 2023) designs a feature extraction backbone consisting of depthwise convolution, pointwise convolution, and transformer self-attention, which can extract local information and learn global dependency at the same time. STARK (Yan et al., 2021) employs a transformer structure to capture spatial and temporal relation between features from continuous frames.

Occlusion is a difficult challenge for visual tracking, and some work attempts to solve this problem. Part-based robust tracking (Yao et al., 2016) and Robust object tracking (He et al., 2016) divide search images into several subregions and utilize the response scores of these subregions and their spatial relationship to locate the target. This sparse pitch strategy might find the corresponding local feature when the target is partially occluded, but it also brings a large computational burden and unsatisfied accuracy of bounding box regression. Learning regression and verification networks (Zhang et al., 2021) and Reliable re-detection (Wang N. et al., 2018) incorporated a detection component to verify if tracking results include the target object, and use it to determine the next search range. It should be noted that this is an auxiliary method and does not necessarily improve the recognition ability of the network of occluded targets. KYS (Bhat et al., 2020) encodes the object information in the background, propagates and updates these encoded vectors in the continuous sequence, and combines them with the target appearance model to locate the target. SINT++ (Wang X. et al., 2018) notices the imbalance in the number of occluded samples within the training dataset and employs reinforcement learning to generate occluded images to enhance the diversity of the training samples. However, this approach requires a high computational cost, which can increase the training time significantly when dealing with large amounts of training data. SiamON (Fan et al., 2021) designs multiple masks manually to generate occlusion samples. This method results in limited occlusion information that does not correspond to real-world scenarios.

## 3 Methods

### 3.1 The overview of the target-aware transformer network

**Figure 2:**
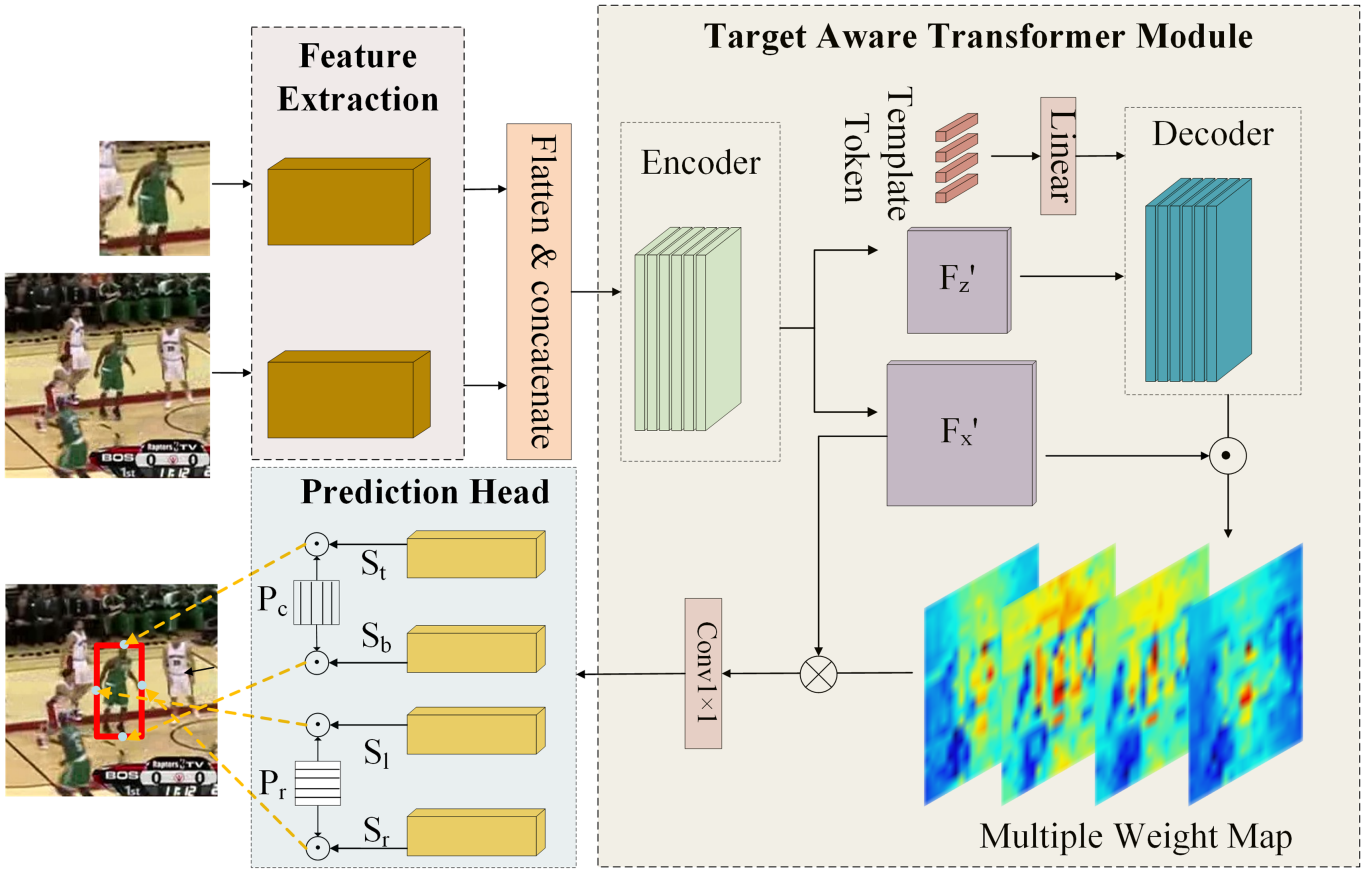
The framework of target-aware transformer network. It consists of three key components, two branches of backbone, a target-aware transformer module, and a boundary prediction head.

We present an innovative network architecture designed for occlusion problems. The structure of the target-aware transformer network is shown in Figure 2. The framework consists of three main components: two branches of feature extraction backbones with shared parameters, a transformer-based feature augment network, and a prediction head.

#### 3.1.1 Backbone

We employ RestNet50 as the feature extraction network with its fully connected layer removed. To preserve the depth of the backbone while considering computation efficiency, we adjust the dilation and channel number of the last convolution block, resulting in a final stride of 16 and an output feature channel of 1,024. A 1 × 1 convolutional layer is added at the end of the backbone to reduce the number of feature channels to 256. The input of the backbone is a template image $P_{z}\in R^{3\times H_{z}\times W_{z}}$ and a search image $P_{x}\in R^{3\times H_{x}\times W_{x}}$ . The template image is cropped from the initial frame based on the given bounding box, and the search image is cropped from the current frame based on the last tracking result. After feature extraction, the template feature $F_{z}\in R^{C\times \frac{H_{z}}{16}\times \frac{W_{z}}{16}}$ and search feature $F_{x}\in R^{C\times \frac{H_{x}}{16}\times \frac{W_{x}}{16}}$ are flattened in spatial dimension following the requirement of transformer and then concatenated as one vector to serve as the input for target-aware transformer module.

#### 3.1.2 Target-aware transformer module

The purpose of the target-aware transformer module is to exploit the reliable appearance information of the target in the template to enhance the search feature. Even if the target is occluded, it can highlight the remaining parts of the target, thereby helping the subsequent head network to make correct predictions. As shown in Figure 2, the target-aware transformer module is a feature fusion network based on the encoder–decoder network. The input is the concatenated feature extracted from the backbone and fed into the encoder directly. The encoder facilitates the global interaction between search and template features, utilizing shared target information for feature enhancement. The encoder can be formulated as:


(1) $$F_{e}=\mathrm{MultiHead}\left(\mathrm{Concat}\left(F_{z},F_{x}\right)\right)$$

where *F_e_* is the output of the encoder; *F_z_, F_x_* is the template and search feature extracted from the backbone; *Concat* is the concatenation operation; and *MultiHead* is multi-head attention. The multi-head attention can be formulated as:


(2) $$\mathrm{MultiHead}\left(Q,K,V\right)=\mathrm{Concat}\left(\mathrm{Attentio}n_{1},\ldots ,\mathrm{Attentio}n_{h}\right)W^{O}$$

(3) $$\mathrm{Attention}\left(Q,K,V\right)=\mathrm{soft}\max\left(\frac{\left(QW_{i}^{Q}\right)\;{\left(KW_{i}^{K}\right)}^{T}}{\sqrt{d_{k}}}\right)\;\left(VW_{i}^{V}\right)$$

where *h* is the number of attention heads; *W_i_^Q^*, *W_i_^K^*, *W_i_^V^*, and *W^O^* are projection matrices; and *Q*, *K*, and *V* are the feature sequences query, key, and value that are generated from the concatenation feature mentioned above.

Then, the output feature of the encoder is further separated into template feature *F_z_’* and search feature *F_x_’*, and only template feature *F_z_’* is utilized as one of the decoder inputs. This is because search features cannot reliably provide target information when the target is occluded and may even bring interference. We employ multiple template tokens *T_t_* as another input for the decoder. The decoder prompts each token to independently learn the distinctive relationship of the target from template features, which can help the network infer the target location from unoccluded parts in the search image. The decoder can be formulated as:


(4) $$F_{d}=\mathrm{MultiHead}\left(\left(T_{t1},T_{t2},\ldots ,T_{tn}\right),{F_{z}}^{'}\right)$$

where *F_d_* is the output of the decoder; *n* is the number of target tokens and we adopted 4 in this algorithm; and *MultiHead* is multi-head attention. The query in this multi-head attention is generated from template tokens *T_t_*, while the key and value are generated from template feature *F_z_’*.

The output of the decoder *F_d_* is dot-multiplied with the search feature *F_x_’* to obtain multiple weight maps, which represent the importance of features in spatial dimension. These multiple weight maps are element-wise multiplied with the search feature *F_x_’* to selectively enhance the features of interest. This calculation process can be formulated as:


(5) $$F=\left(F_{d}⊙{F_{x}}^{'}\right)⊗{F_{x}}^{'}$$

where *F* is the enhanced feature.

At last, the enhanced features *F* are compressed in channel dimension through a 1 × 1 convolution layer to generate the final output of the target-aware transformer module.

**Figure 3:**
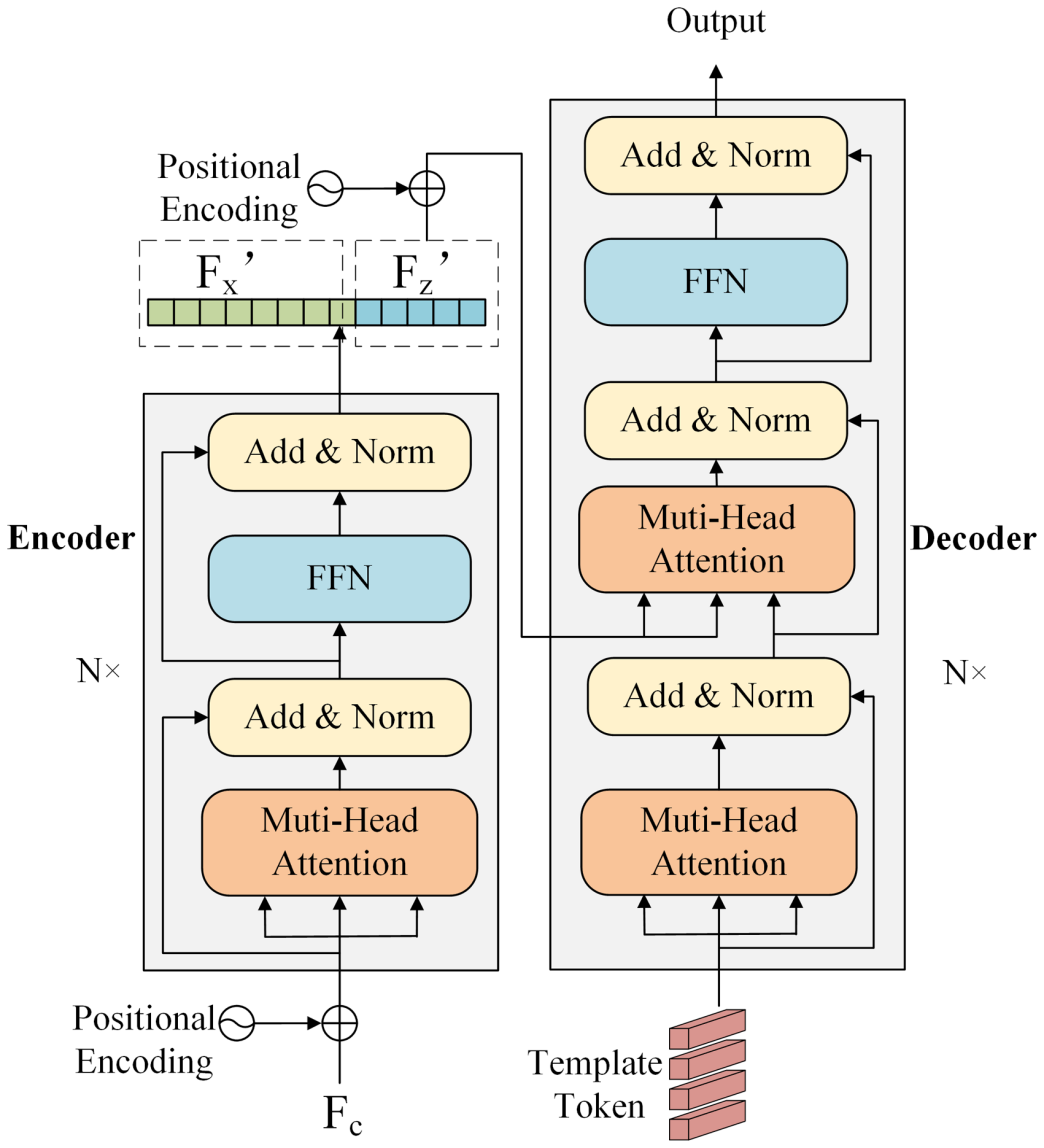
The structure of the encoder and decoder network, including N encoder layers and N decoder layers, respectively.

The specific structure of the encoder and decoder network is shown in Figure 3. The encoder comprises 6 encoder layers that share the same network structure. The encoder layer contains a multi-head attention component, a feed-forward network (FFN), and two residual components. The input feature *F_c_* is added to sine-cosine position encoding to embed position information. The encoder learns the correlation between each element of the input feature. The decoder consists of six decoder layers with the same structure, which contains two multi-head attention components, a feed-forward network, and three residual components. The decoder first performs self-attention on template token input and then establishes the information interaction between it and the template feature *F_z_’*.

#### 3.1.3 Boundary prediction head

TransT (Chen et al., 2021) employs two branches of three-layer perceptions to predict the center point and corresponding size of the bounding box, which will cause the Dirac distribution fitting problem (Li et al., 2020). To address this, STARK (Wang et al., 2023) proposes a head network to predict the top-left and bottom-right corners of the target bounding box. Nevertheless, these two points are typically located outside the target and lack strong target appearance features, and the prediction results can be easily affected by occlusion or distractor covering either corner point. Therefore, we propose a novel prediction head to generate accurate bounding boxes for the occluded target. It consists of four independent branches that directly predict the four boundaries of the bounding box instead of being limited to specific points. Each branch of the prediction head is composed of 5 sets of 1 × 1 convolutional layer, BN layer, ReLU activation function, and an extra 1 × 1 convolutional layer. The output features of the target-aware transformer module are fed into the head network to generate the boundary probability maps *S_t_*, *S_b_*, *S_l_*, *S_r_*, where the feature’s spatial scale remains unchanged and the channel dimension is compressed to 1. Then, the probability maps are aggregated with the spatial position *P_c_*, *P_r_* in horizontal and vertical directions, respectively, to obtain the four boundaries of the predicting bounding box *y_t_*, *y_b_*, *x_l_*, *x_r_*. The aggregation process of probability maps and spatial position is formulated as:


(6) $$y_{t}=\overset{H}{\underset{y=0}{\sum }}\overset{W}{\underset{x=0}{\sum }}P_{c}\left(x,y\right)\cdot S_{t}\left(x,y\right)$$

(7) $$y_{t}=\overset{H}{\underset{y=0}{\sum }}\overset{W}{\underset{x=0}{\sum }}P_{c}\left(x,y\right)\cdot S_{t}\left(x,y\right)$$

(8) $$x_{l}=\overset{H}{\underset{y=0}{\sum }}\overset{W}{\underset{x=0}{\sum }}P_{r}\left(x,y\right)\cdot S_{l}\left(x,y\right)$$

(9) $$x_{r}=\overset{H}{\underset{y=0}{\sum }}\overset{W}{\underset{x=0}{\sum }}P_{r}\left(x,y\right)\cdot S_{r}\left(x,y\right)$$

### 3.2 Hard occlusion instance generation module

**Figure 4:**
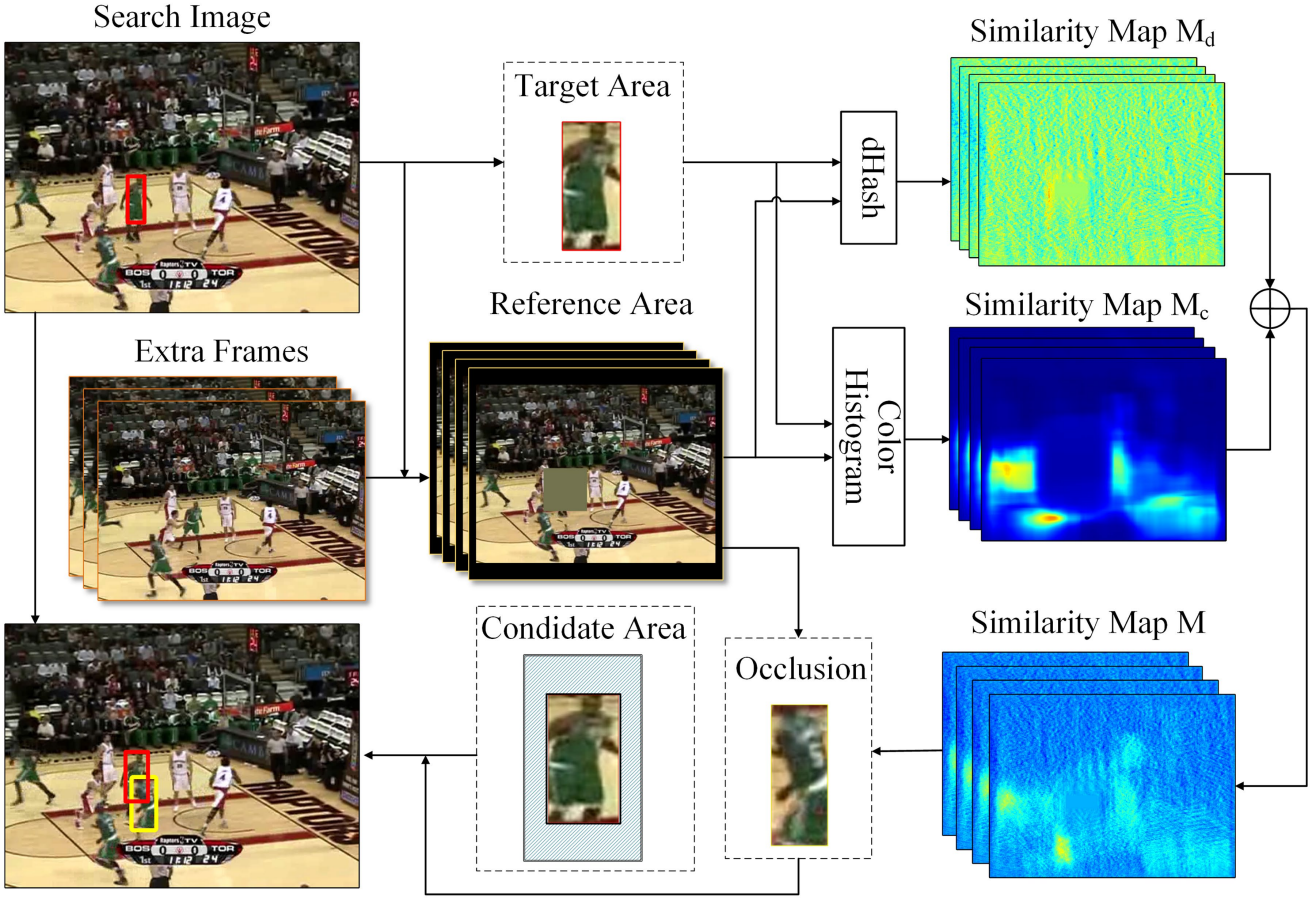
The structure of the hard instance generation module.

We design a hard occlusion instance generation module to address the issue of lacking occlusion training samples. We select regions in the background with similar appearance information to the target as the occlusion. It can help the network learn to recognize incomplete target appearance information and enhance the distinguishing capability of the target at the same time. The structure of the proposed module is shown in Figure 4. Firstly, the target area $R_{t}\in R^{3\times H_{t}\times W_{t}}$ is cropped from the search image $P\in R^{3\times H\times W}$ based on the ground truth, while the reference area $R_{r}\in R^{3\times \left(H_{t}+H\right)\times \left(W_{t}+H\right)}$ is generated by masking the target region in the search image and then padding based on the target size. We generate extra reference area from several frames within the same video sequence to avoid the issue that the current frames contain no similar region to the target. A sliding window approach is utilized within the reference area to generate H×W sub-reference regions $R_{s}\in R^{3\times H_{t}\times W_{t}}$ of the same size as the target area. Subsequently, the similarity map $M_{d}\in R^{H\times W}$ is obtained by calculating the Hamming distance between the DHash of the target area and each subregion. It can be formulated as:


(10) $$M_{d\left(i,j\right)}=D\left(H_{d}\left(R_{t}\right),H_{d}\left(R_{r\left(i,j\right)}\right)\right)$$

where *D* is the Hamming distance, and *H_d_* is DHash which generates a binary numerical representation of an image by comparing the pixel values of adjacent pixels.

Another similarity map $M_{c}\in R^{H\times W}$ is obtained by calculating the correlation coefficient between the color histogram of the target area and each subregion, which can be formulated as:


(11) $$M_{c\left(i,j\right)}=C\left(H_{c}\left(R_{t}\right),H_{c}\left(R_{r\left(i,j\right)}\right)\right)$$

where *C* is the correlation coefficient, and *H_c_* is the color histogram.

These two maps are then element added to generate the final similarity map $M\in R^{H\times W}$ . The calculation process of the similarity map *M* can be formulated as:


(12) $$M=w_{1}M_{d}+w_{2}M_{c}$$

where *w_1_* and *w_2_* are weight parameters, we adopt *w_1_* = 0.33 and *w_2_* = 0.67.

Based on the experimental results provided by DeVries and Taylor (2017), the shape of occlusions is not a significant factor when the generation location is random. Therefore, the occlusion $R_{o}\in R^{3\times H_{t}\times W_{t}}$ is cropped from the reference area using the coordinate of the highest similarity score position as the center and the size of the target area. To prevent excessive occlusion of the target region, which may result in insufficient target information for the network, or occlusion that is too small to effectively occlude the target, we establish a candidate area around the target region by setting the intersection over union $IOU\in \left[m,n\right]$ (*m, n* are threshold parameters, we set *m* = 0.125, *n* = 0.600) of the occlusion area and the target region. A point is randomly chosen as the center coordinate of the occlusion in the candidate area, limiting the occluded area of the target region to a suitable range. Specifically, the hard occlusion instance generation module does not operate on every training sample, but rather based on a certain probability *p*, we adopt *p* = 0.01.

## 4 Experiments

### 4.1 Implementation details

#### 4.1.1 Offline training

We chose COCO (Lin et al., 2014), LaSOT, TrackingNet, and Got10k to form the training dataset. A pair of images are selected at random intervals from the same video sequence as the input of the network. These pairs undergo image enhancement including brightness jitter and horizontal flip, before being cropped to sizes of 128 × 128 and 320 × 320 for the template and search images, respectively. The target jitter is carried out in search images to avoid center bias. The parameters of ResNet50 have been pretrained on ImageNet, while the remaining parameters of the network are initialized through Xavier Uniform. We utilized AdamW as the optimizer to train the network, with a learning rate of 1e − 5 for the backbone and 1e − 4 for the other parts, and a weight decay of 1e − 4. There are 700 epochs during the entire training process, each consisting of 50,000 iterations. The learning rate would decrease by a factor of 10 once the epoch exceeded 400. The loss function is a linear combination of $l_{1}$ loss and GIoU loss, it is written as follows:


(13) $$L=\alpha L_{1}+\beta L_{\mathrm{GIoU}}$$

where α , β are hyperparameters, we set α =5, β =2.

#### 4.1.2 Inference process

During the online tracking process, the template is cropped from the first frame of each video sequence, and its feature remains fixed throughout subsequent frames. The search image is cropped in the current frame based on the tracking result from the previous frame. The predicted bounding box output by the network does not undergo spatial modulation, such as the Hanning window penalty, which avoids the hyperparameters adjustment. We add a validation mechanism for the tracking results to prevent the bounding box from drifting to the obvious wrong area when the target disappears or the tracker fails. This is achieved by computing the correlation between the feature within the predicted region and the template feature. If the correlation falls below the set threshold, the search area will be expanded in the next frame.

### 4.2 Evaluation results on several datasets

In this section, we evaluate the performance of our tracker on five datasets, including LaSOT, TrackingNet, Got10k, OTB100, and UAV123 and compare it with several state-of-the-art algorithms, including TransT, STARK, CSWinTT, MixFormer, ToMP, KeepTrack (Mayer et al., 2021), TrDiMP (Wang et al., 2021), SiamBan, SiamRPN++, ATOM (Danelljan et al., 2019), TrTr, SiamFC++, KYS, SBT (Xie et al., 2022), Ocean (Zhang et al., 2020), DiMP (Bhat et al., 2019), and SiamAttn. The results demonstrate the effectiveness of our approach.

#### 4.2.1 LaSOT

**Figure 5:**
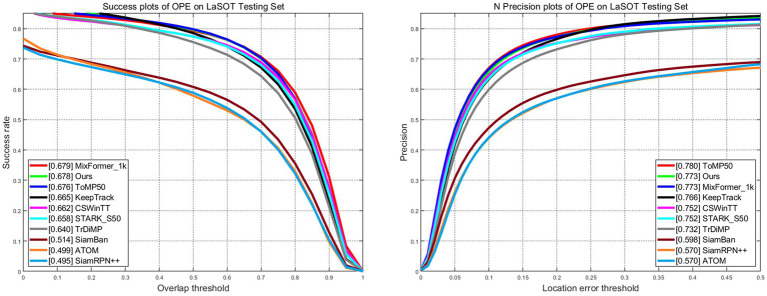
Success and norm precision plots on LaSOT.

**Figure 6:**
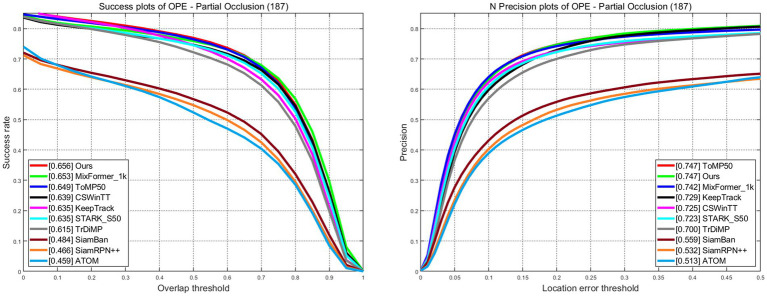
Success and norm precision plots with partial occlusion on LaSOT.

**Figure 7:**
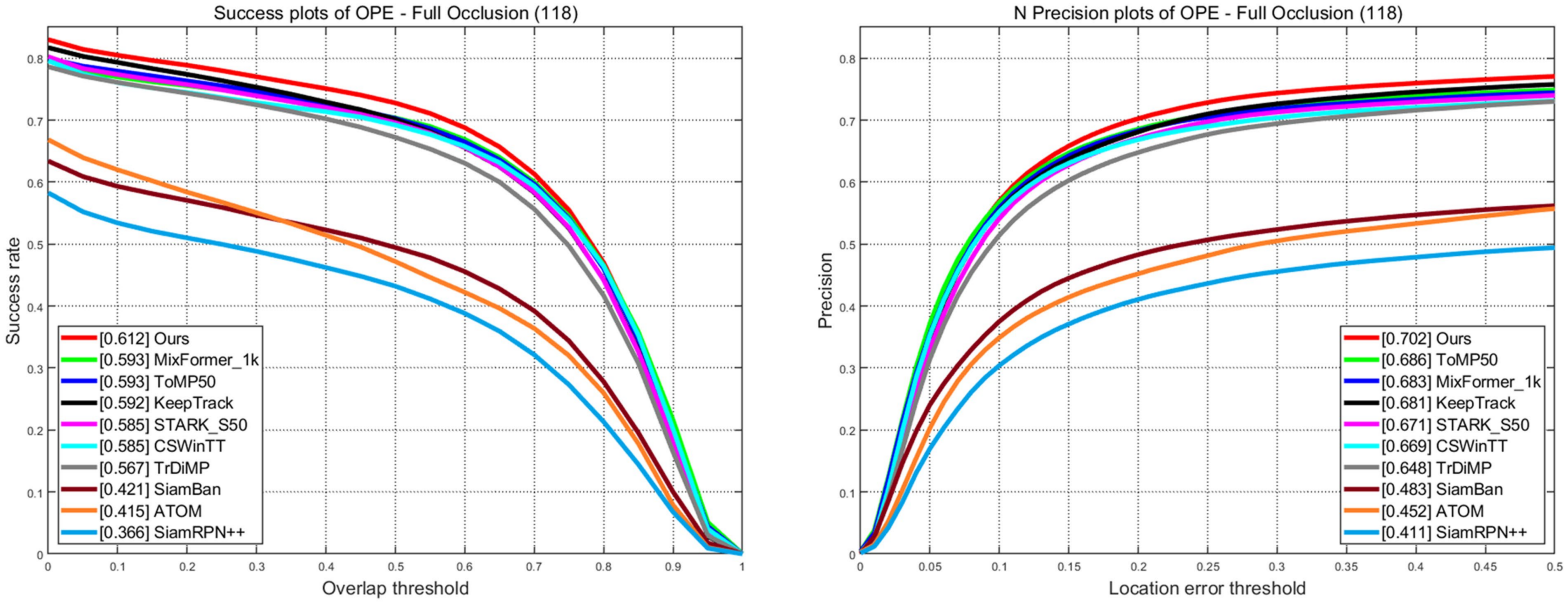
Success and norm precision plots with full occlusion on LaSOT.

LaSOT is a large-scale dataset that provides long-term tracking sequences. It consists of 1,400 video sequences, out of which 1,120 are used for training and 280 for testing. The average length of the video sequences exceeds 2,500 frames. The success plots and norm precision are shown in Figure 5. Our tracker demonstrates excellent performance with a success rate of 67.8% and a norm precision rate of 77.3%. Our tracker ranks first in both success rate and precision rate compared with trackers that also adopt CNN + Transformer architecture. The success rate is only 0.1% lower than Mxformer-1 k, a tracker with a transformer backbone that leads to high training costs and slow real-time tracking speeds. Our tracker outperforms the STARK-S50 by 2% in success rate and 2.1% in precession rate. The success plots and precession plots with partial occlusion and full occlusion are shown in Figures 6, 7. Our tracker takes first place in both occlusion circumstances, with a gain of 2.1 and 2.7% in AUC compared with STARK-S50. This indicates that our proposed algorithm provides significant promotion in addressing occlusion issues.

#### 4.2.2 TrackingNet

TrackingNet is a large-scale tracking dataset that offers over 30,000 video sequences sourced from YouTube, featuring a diverse range of real-world scenarios and object categories. The testing part comprises 511 videos, and the tracker’s performance is evaluated by uploading the tracking results to the server for online assessment. Table 1 presents the evaluation scores AUC, precision, and norm precision. Our tracker achieves an AUC of 82.2% and a norm precision of 86.9%, which exceeded that of STARK-S50 at 1.9% and 1.8%, respectively. Our tracker takes first place both in AUC and norm precision among trackers that also employ the CNN + Transformer structure.

**Table 1:** Experiment results on TrackingNet, Got10k, and VOT2018.

Method	TrackingNet			Got10k			VOT2018		
	AUC (%)	P_Norm_ (%)	P (%)	AO (%)	SR_0.5_ (%)	SR_0.75_ (%)	Accuracy	Robust	EAO
MixFormer	82.6	87.7	81.2	71.2	79.9	65.8	-	-	-
CSWinTT	81.9	86.7	79.5	69.4	78.9	65.4	-	-	-
ToMP50	81.2	86.2	78.6	-	-	-	-	-	-
TransT	81.4	86.7	80.3	67.1	76.8	60.9	-	--	-
STARK-S50	80.3	85.1	80.0	67.2	76.1	61.2	-	-	-
SwinTrack	82.5	87.0	80.4	69.4	78.0	64.3	-	-	-
TrDiMP	81.2	85.4	78.4	67.1	77.7	58.3	0.601	0.141	0.462
TrTr	69.3	77.2	-	-	-	-	0.612	0.234	0.424
KYS	74.0	80.0	68.8	63.6	75.1	51.5	0.609	0.143	0.462
SiamFC++	75.4	80.0	70.5	59.5	69.5	47.9	0.587	0.183	0.426
SiamRPN++	73.3	80.0	69.4	51.7	61.6	32.5	0.600	0.234	0.414
ATOM	64.8	77.1	70.3	-	-	-	0.590	0.204	0.401
Ocean	-	-	-	59.2	69.5	-	0.598	0.169	0.467
SiamBan	-	-	-	-	-	-	0.597	0.178	0.452
SiamAttn	75.2	81.7	-	-	-	-	0.630	0.160	0.470
SBT	-	-	-	69.9	80.4	63.6	-	-	-
Ours	82.2	86.9	80.4	69.1	78.2	64.1	0.612	0.169	0.458

#### 4.2.3 Got10k

Got10k is a large-scale tracking dataset that consists of over 10,000 video sequences, encompassing 560 object classes and 87 motion patterns. The tracking results should be uploaded to the online server for evaluation. It is required that the tested tracker can only be trained on the Got10k training set, and we have adhered to this requirement. As reported in Table 1, our tracker outperforms the baseline tracker STARK-S50 with AO increased by 1.9% and SR_0.5_ increased by 2.1%.

#### 4.2.4 VOT2018

VOT2018 is a commonly used short-term dataset for evaluating tracking performance, consisting of a total of 60 video sequences. VOT introduces a restart mechanism that allows trackers to be re-initialized after tracking failures, thereby improving the utilization of video sequences. The evaluation of VOT2018 includes accuracy, robust, and EAO. As shown in Table 1, our tracker achieves a 0.612 accuracy score, 0.169 robust score, and 0.459 EAO, which is a satisfactory result among recent algorithms.

#### 4.2.5 OTB100

OTB100 consists of 100 video sequences, representing 11 challenging tracking scenarios. The evaluation results are shown in Table 2, and our tracker achieves an AUC of 70.1% surpassing the baseline tracker STARK-S50 1.8%.

**Table 2:** Experiment results on OTB100, UAV123, and NFS in terms of AUC.

	SiamRPN++	ATOM	KeepTrack	TrDiMP	TransT	KYS	STARK-S50	ToMP50	SBT	DiMP	Ours
OTB100	69.6	66.7	70.9	71.1	69.4	69.5	68.3	70.1	70.9	62.0	70.1
UAV123	61.3	64.2	69.7	67.5	69.1	65.0	69.1	69.0	-	65.3	69.3
NFS	-	59.0	66.4	66.2	65.7	63.5	64.3	66.9	-	62.0	66.1

#### 4.2.6 UAV123

The UAV23 contains 123 videos captured by drones at low altitudes, characterized by frequent changes in view angle and small target sizes. The evaluation results are shown in Table 2, and our method achieves a competitive AUC score compared with state-of-the-art transformer-based trackers.

#### 4.2.7 NFS

NFS includes 100 video sequences with fast moving targets. We evaluate our tracker on the 30 FPS version of NFS. As shown in Table 2, our tracker obtains an AUC score of 66.1%, which outperforms STARK-S50 by 1.8%.

### 4.3 Ablation study

The ablation experiment is conducted on the LaSOT dataset to analyze the impact of different components in the proposed network on tracking performance. The network of STARK-S50 is considered a baseline model with the same ResNet50 backbone. As shown in Table 3, the baseline algorithm achieves an AUC score of 65.8% and a P_nrom_ score of 75.2. After utilizing the target-aware transformer module (TATM), the AUC score improves to 66.6% and the P_nrom_ score increases to 76.4%. Furthermore, with the application of boundary prediction head (BPH), both UAC and P_nrom_ scores are further enhanced by 0.5% and 0.2%, respectively. The employment of a hard occlusion instance generation module (HOIM) during the training process results in the highest performance of the tracker, with an AUC score of 67.8% and a P_nrom_ score of 77.3%. The ablation experiments demonstrate that the proposed target-aware augment module and boundary prediction head, as well as the hard occlusion sample generation module can enhance tracking performance and their collaboration brings positive effects.

**Table 3:** Ablation experimental results on LaSOT.

	HOIG	TATM	BPH	AUC	P_norm_
1				65.8	75.2
2		√		66.6	76.4
3		√	√	67.1	76.6
4	√	√	√	67.8	77.3

**Figure 8:**
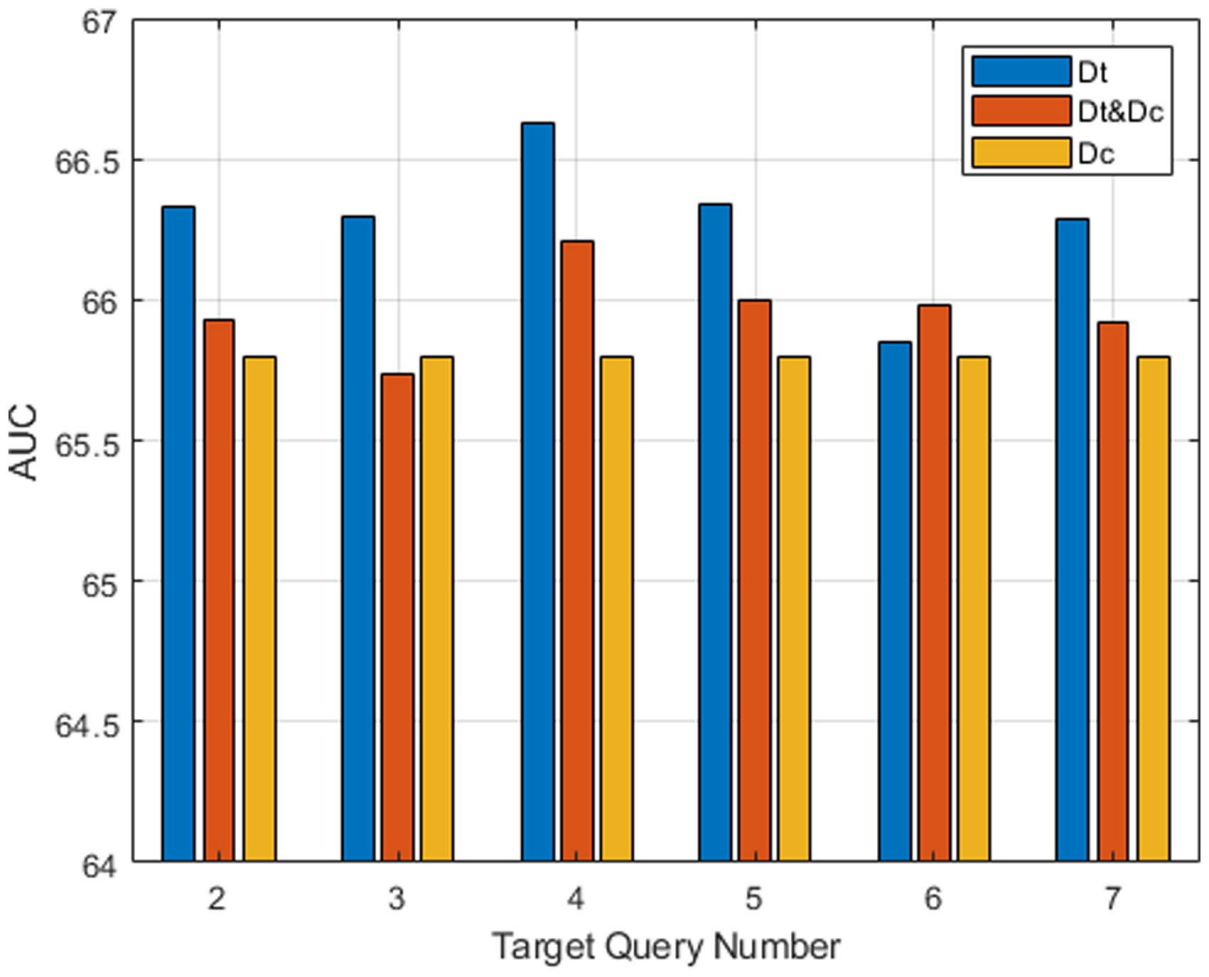
Ablation study of TATT structure on LaSOT.

We also conduct experiments on three structures of the feature fusion module to validate the reasonability of our version. The first one is the proposed target-aware augment module, whose decoder *D_t_* only takes template features and multiple target queries as input. The second one is from STARK-S50, whose decoder *D_c_* takes a query and a feature concatenated from the template and search feature as input. The last one contains both decoders *D_t_ & D_c_*, and the embedding features are concatenated along channel dimension and then pass through a 1 × 1 convolution layer. Figure 8 illustrates the AUC scores of three version modules with varying target query numbers. The tracker with both decoders *D_t_&D_c_* outperforms the one with only *D_c_* at each target query number, and our version with only *D_t_* exhibits the best overall performance. This indicates that the target information embedded within the template feature is more reliable than that of the search feature, even when the target undergoes appearance deformation during the tracking process. Notably, our target-aware augment module achieves the highest AUC score when the target query number is four.

## 5 Conclusion

In this article, we propose target-aware transformer tracking with hard occlusion instance generation to address the issue of tracking failure in occlusion scenes due to the network’s inability to recognize incomplete target appearance information. We propose a target-aware transformer network and a hard occlusion instance generation module. The hard occlusion instance generation module selects an image patch similar to the target from images within the same video sequence as occlusion and then randomly overlays around the target area in the search image to generate occlusion training samples. This method can help the network learn to recognize incomplete target appearance information and enhance the distinguishing capability of the target without adding an extra network. The target-aware transformer network is built on transformer architecture, which can facilitate global interaction between template and search features and utilizes template features to generate embedding vectors to selectively enhance search features. The intersection between the target and background can be directly predicted by the head network, which enables the full utilization of unobstructed target information to generate tracking boxes. The proposed tracker is evaluated on five commonly used tracking benchmarks: LaSOT, TrackingNet, Got10k, OTB100, and UAV123 against several state-of-the-art trackers. Our trackers show outstanding performance, especially in the occlusion category. The ablation experiments show the positive effect of the proposed network and occlusion instance generation module. Tracking in the full occlusion scenes remains a challenge. The tracking box may be drawn toward the background, causing the target to go beyond the search range and resulting in tracking failure. In future work, a detection mechanism can be incorporated into the tracking algorithm to verify the tracking results. When the tracking target is deemed lost, it can be recaptured using the detection results and spatial location information.

## Data availability statement

Publicly available datasets were analyzed in this study. This data can be found at: Visual Tracker Benchmark (hanyang.ac.kr), LaSOT—Large-scale Single Object Tracking (stonybrook.edu), GOT-10 k: Generic Object Tracking Benchmark (aitestunion.com), TrackingNet (tracking-net.org), A Benchmark and Simulator for UAV Tracking (Dataset) | IVUL | Image and Video Understanding Lab (kaust.edu.sa).

## Author contributions

DX: Formal analysis, Investigation, Methodology, Software, Writing – original draft. ZW: Data curation, Funding acquisition, Writing – review & editing. GZ: Project administration, Supervision, Writing – review & editing.
